# An Attacker–Defender Game Model with Constrained Strategies

**DOI:** 10.3390/e26080624

**Published:** 2024-07-24

**Authors:** Jiaqi Ren, Jin Liu, Yibo Dong, Zhe Li, Weili Li

**Affiliations:** National Key Laboratory of Information Systems Engineering, National University of Defense Technology, Changsha 410073, China; jiaqiren@nudt.edu.cn (J.R.); liujin229234@nudt.edu.cn (J.L.); dongyibo@nudt.edu.cn (Y.D.); lizhe@nudt.edu.cn (Z.L.)

**Keywords:** infrastructure attack and defense scenarios, complex networks, game theory, constrained strategies, information theory

## Abstract

Recently, research interest in the field of infrastructure attack and defense scenarios has increased. Numerous methods have been proposed for studying strategy interactions that combine complex network theory and game theory. However, the unavoidable effect of constrained strategies in complex situations has not been considered in previous studies. This study introduces a novel approach to analyzing these interactions by including the effects of constrained strategies, a factor often neglected in traditional analyses. First, we introduce the rule of constraints on strategies, which depends on the average distance between selected nodes. As the average distance increases, the probability of choosing the corresponding strategy decreases. Second, we establish an attacker–defender game model with constrained strategies based on the above rule and using information theory to evaluate the uncertainty of these strategies. Finally, we present a method for solving this problem and conduct experiments based on a target network. The results highlight the unique characteristics of the Nash equilibrium when setting constraints, as these constraints influence decision makers’ Nash equilibria. When considering the constrained strategies, both the attacker and the defender tend to select strategies with lower average distances. The effect of the constraints on their strategies becomes less apparent as the number of attackable or defendable nodes increases. This research advances the field by introducing a novel framework for examining strategic interactions in infrastructure defense and attack scenarios. By incorporating strategy constraints, our work offers a new perspective on the critical area of infrastructure security.

## 1. Introduction

Currently, infrastructure networks, such as water supply networks, aviation networks, and transportation networks, play essential roles in human society [1,2]. Excessive dependency on these networks results in human systems having a wide range of vulnerabilities, including to the threats posed by both terrorists and hackers. For example, the September 11 attacks against the World Trade Center in New York and the Pentagon in Virginia resulted in a significant loss of life and had an enormous impact on the economy and politics. Moreover, networks are also prime targets during times of conflict. Therefore, it is vital to consider adversaries’ strategies and understand network interdependencies from a global perspective.

Numerous methods, such as probabilistic risk analyses and data analyses, have been proposed to protect infrastructures [3,4]. These methods are unsuitable for modeling the behavior of intelligent adversaries [3,4,5]. In these cases, game theory provides an appropriate model framework to address this problem, within which the optimal strategies and interactions of players can be assessed [6,7]. For example, Brown et al. [8] formulated a sequential game model to minimize the operating costs for both attack and defense strategies. Pita et al. [9] employed game theory to examine the complexity of airport security. Zhang et al. [10] proposed a game model to address challenges in factory safety management. Feng et al. [11] took this a step further by integrating game theory and risk assessments to evaluate protective measures for multiple chemical facilities under the looming threat of attacks. They later expanded their study to incorporate multiple attackers [12]. Zhang et al. [13] investigated resource allocation within security games, while Guan et al. [14] delved into an attack–defense game model that incorporated budget constraints. Zhang et al. [15] transformed the game of an infrastructure problem into a multiobjective optimization model and employed evolutionary algorithms to solve it.

However, importantly, the above studies overlook the complex interactions that exist within infrastructure systems. In reality, interconnected infrastructures form a complex network, wherein the failure of a single facility could potentially affect the entire network. A typical network consists of nodes, edges connecting the nodes, and weights assigned to the edges. Initially, mathematicians believed that real systems could be represented by regular structures such as regular lattices and nearest-neighbor grids. By the late 1950s, Erdős et al. [16] introduced random networks, in which the existence of an edge between two nodes is determined by a probability. Networks generated via this method are referred to as random networks. In recent decades, research on small-world networks and scale-free networks has initiated complex network studies. Watts et al. [17] proposed a small-world network model involving the rewiring of the edges between nodes in a regular network. Barabási et al. [18] introduced the scale-free network model, which is characterized by a few nodes with many connections, resulting in a power-law distribution of the node degrees in this type of network. Li et al. [19] proposed a localized world evolutionary network model by using the world trade web. Comellas [20] introduced a small-world network model with a certain regularity in its node connections from the perspective of graph theory to study the topology of communication networks.

Therefore, it is crucial to consider the comprehensive impact of localized infrastructure failures on the entire infrastructure network. To address this issue, protection measures for infrastructure networks should be analyzed by integrating game theory and complex network theory. Fu et al. [21] developed a static network attack and defense game model to examine the impact of cascading failures. Gu et al. [22] analyzed the significance of the Bayesian Stackelberg game model from the perspective of network science. Zeng et al. [23] used the Bayesian Stackelberg game model and proposed a false network construction method. Qi et al. [24,25] proposed a link-hiding rule and analyzed its optimization impact within the context of dynamic attack and defense games in complex networks. Huang et al. [26] used sequential game theory to model attack and defense games in complex networks and proposed a strategy optimization method. Baykal-Guersoy et al. [27] introduced the concept of an attack number, which considers factors such as the number of affected individuals or the occupancy level of critical infrastructure, as a measurement. They developed a game model to examine the security of transportation networks. Li et al. [28,29,30] proposed an attack–defense model that takes a network perspective to investigate how network structure and cost constraints influence equilibrium outcomes under two typical strategies. Thompson et al. [31,32] analyzed the potential impacts of intelligent attacks and worst-case interruptions on the U.S. air transportation network. Subsequently, they established a defender–attacker–defender optimization model with three levels and proceeded to solve it. These game models can be roughly divided into two categories. One category is that of the simultaneous game models, where the attacker and the defender do not know their opponent’s chosen strategies [28,29,33]. The other category, containing the Stackelberg (sequential) game models, is the one in which the attacker can effectively surveil the security measures of the defender [23,24,25,34,35,36].

In these studies, it is assumed that players’ strategies are not constrained, which is not always possible in realistic situations. In practice, players are often restricted by objective conditions when choosing strategies. Charnes [37] developed the two-person zero-sum constrained game. Owen [38] investigated the existence of solutions to the two-person zero-sum constraint matrix countermeasure problem using dual linear programming theory. Firouzbakht et al. [39] proposed a constrained bimatrix game framework that has practical applications in various fields, such as modeling packet jamming in wireless networks. Xiao et al. [40] proposed an interval bimatrix game with a constrained strategy.

In this paper, we introduce a new approach to the attacker–defender game model by incorporating strategy constraints. Considering the average distances between nodes is crucial for securing critical infrastructures like high-speed rail (HSR) networks. Shorter distances between stations enable the rapid communication of security signals, essential for swift detection and responses to threats. This quick communication directly impacts the response time of automated security measures. During an attack, shorter distances can significantly reduce the time needed to activate security protocols, mitigating the attack’s severity. By incorporating the average node distance as a key metric, our model introduces a method for quantifying the feasibility of strategy selection. The larger the average distance between selected nodes, the more difficult it is to apply that strategy in realistic situations. This approach is not only innovative within the field but also mirrors practical scenarios. We conduct experiments in a target network to analyze the impacts of these constraints.

The rest of the paper is organized as follows: In Section 2, we present some basic assumptions, constrained strategies, and payoffs. In Section 3, the method used for solving the game is presented. The equilibrium results are analyzed in Section 4. Finally, Section 5 concludes the paper.

## 2. An Attacker–Defender Game Model Based on Constrained Strategies

Considering constrained strategies, we build an attacker–defender game model for infrastructure networks. An infrastructure network can be represented by an undirected simple graph G(V,E), where $V=\left\{V_{1},V_{2},\cdots ,V_{N}\right\}$ represents the node set, N=|V| is the number of nodes, and $E=\left(e_{ij}\right)\subseteq V\times V$ represents the link set. Let the adjacency matrix of graph *G* be $A\left(G\right)={\left(a_{ij}\right)}_{N\times N}$. If there is a link between nodes $V_{i}$ and $V_{j}$, then $a_{ij}=a_{ji}=1$; otherwise, $a_{ij}=a_{ji}=0$.

Since the actions of the attacker and the defender are simultaneous, this game model is a static model. The attacker–defender game model uses a ten-tuple to represent the confrontation, where $ADG=\left(N_{A},N_{D},V^{A},S_{A},V^{D},S_{D},P_{A},P_{D},U_{A},U_{D}\right)$:

(1) Let $N_{A}$ represent the attacker in the attacker–defender game model. The attacker predicts the defense strategy of the defender to develop an attack strategy.

(2) Let $N_{D}$ represent the defender in the attacker–defender game model. The defender predicts the attack strategy of the attacker to develop a defense strategy.

(3) Let $V^{A}$ represent the attack node set. If the attacker chooses to target nodes $V_{1}$ and $V_{2}$, then $V^{A}=\left\{V_{1},V_{2}\right\}$.

(4) Let $S_{A}=\left\{S_{A}^{1},S_{A}^{2},\cdots ,S_{A}^{i},\cdots ,S_{A}^{m}\right\}$ represent the attack strategy set. The vector $S_{A}^{i}=\left[x_{1},x_{2},\cdots ,x_{N}\right]$ indicates the *i*th attack strategy in the set of attack strategies. In this case, $x_{i}=1$ if the $V_{i}$ node is attacked ($V_{i}\subseteq V^{A}$); otherwise, $x_{i}=0$.

(5) Let $V^{D}$ represent the defense node set. If the defender chooses to target nodes $V_{3}$ and $V_{4}$, then $V^{D}=\left\{V_{3},V_{4}\right\}$.

(6) Let $S_{D}=\left\{S_{D}^{1},S_{D}^{2},\cdots ,S_{D}^{j},\cdots ,S_{D}^{n}\right\}$ represent the defense strategy set. The vector $S_{D}^{j}=\left[y_{1},y_{2},\cdots ,y_{N}\right]$ indicates the *j*th defense strategy in the set of defense strategies. In this case, $y_{i}=1$ if the $V_{i}$ node is defended ($V_{i}\subseteq V^{D}$); otherwise, $y_{i}=0$.

(7) Let $P_{A}=\left\{P_{A}^{1},P_{A}^{2},\cdots ,P_{A}^{i},\cdots ,P_{A}^{m}\right\}$ represent the probability that the attacker adopts an attack strategy. The element $P_{A}^{i}$ indicates that the attacker adopts the $S_{A}^{i}$ strategy with a probability of $P_{A}^{i}$.

(8) Let $P_{D}=\left\{P_{D}^{1},P_{D}^{2},\cdots ,P_{D}^{j},\cdots ,P_{D}^{n}\right\}$ represent the probability that the defender adopts a defense strategy. The element $P_{D}^{j}$ indicates that the defender adopts the $S_{D}^{j}$ strategy with a probability of $P_{D}^{j}$.

(9) Let $U_{A}=U_{A}\left(S_{A},S_{D}\right)$ represent the profit function for the attacker. The value of the function also depends on $S_{A}$ and $S_{D}$. Different attack strategies and different defense strategies generate different profit values for the attacker.

(10) Let $U_{D}=U_{D}\left(S_{A},S_{D}\right)$ represent the profit function for the defender. The value of the function depends on $S_{A}$ and $S_{D}$. Different attack strategies and different defense strategies generate different profit values for the defender.

### 2.1. Basic Assumptions

(1) In this game, there are two rational players, namely, the attacker and the defender. Both players possess complete information about the target network, including knowledge of all possible strategies and the objective metrics associated with the network’s structure for each strategy profile in the network.

(2) All attacks and defenses are target nodes. A node is considered to be successfully attacked when it is targeted by the attacker without being protected by the defender. Once a node is successfully attacked, all the edges connected to that node are removed from the network.

(3) In this game, both the attacker and the defender independently formulate their strategies without prior knowledge of each other’s decisions. This simultaneous move structure is designed to capture scenarios in which each party operates under conditions of strategic secrecy and independent decision-making. Furthermore, the game is structured as a single-shot interaction, implying that there are no subsequent rounds which could provide opportunities for reassessment or adaptation based on an opponent’s previous moves.

### 2.2. Constraint Strategies

The attack strategy’s selection probability is denoted as $P_{A}^{i}$, while the defense strategy’s selection probability is represented by $P_{D}^{j}$. The strategy constraints are established as follows:

(1) $$P_{A}^{i}\leq \alpha_{A}^{i},\;\forall S_{A}^{i}\in S_{A},$$

and

(2) $$P_{D}^{j}\leq \alpha_{D}^{j},\;\forall S_{D}^{j}\in S_{D},$$

where $\alpha_{A}^{i}$ and $\alpha_{D}^{j}$ are constraint coefficients for the attacker and the defender, respectively, and belong to the interval (0,1).

**Figure 1:**
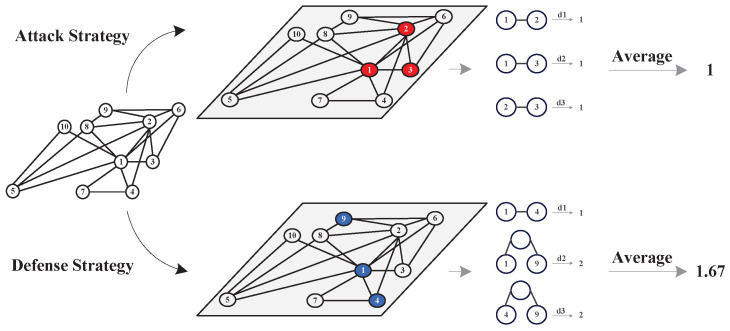
The process of calculating the average distances in this model. The red dots denote the nodes that the attacker chooses to attack and the black dots denote the nodes that the defender chooses to defend.

Figure 1 provides a detailed illustration of the method used for calculating the strategy selection probability based on the average distance between selected nodes. In an example network comprising 10 nodes, we presume that both the attacker and the defender opt for three nodes for their respective strategies. The shortest paths between each pair of nodes are then meticulously calculated. The figure shows the process of deriving the average of these shortest paths, which forms the foundation for imposing constraints on strategy selection.

In this model, as the average distance between selected nodes increases, the probability of choosing their corresponding strategy decreases. We denote the average distance as ${\bar{\mathrm{Dist}}}_{A}^{i}$ for the *i*th attack strategy and ${\bar{\mathrm{Dist}}}_{D}^{j}$ for the *j*th defense strategy. We set the strategy constraint rules as follows:

(3) $$C_{A}^{i}=\frac{\exp\left(\frac{3}{{\bar{\mathrm{Dist}}}_{A}^{i}}\right)}{{\left({\bar{\mathrm{Dist}}}_{A}^{i}\right)}^{3}},\;\forall S_{A}^{i}\in S_{A},$$

and

(4) $$C_{D}^{j}=\frac{\exp\left(\frac{3}{{\bar{\mathrm{Dist}}}_{D}^{j}}\right)}{{\left({\bar{\mathrm{Dist}}}_{D}^{j}\right)}^{3}},\;\forall S_{D}^{j}\in S_{D}.$$

Let $\theta_{A}$ represent the attack strategy constraint parameter and $\theta_{D}$ represent the defense strategy constraint parameter. These parameters indicate the strength of the strategy constraints for the two players. The values of $\theta_{A}$ and $\theta_{D}$ depend on the targeted network structures, the players’ experience, and their subjective preferences. Larger values of $\theta_{A}$ and $\theta_{D}$ indicate weaker constraints, while smaller values indicate stronger constraints. For the attacker, $\alpha_{A}^{i}$ is calculated by

(5) $$\alpha_{A}^{i}=\frac{\theta_{A}\left(C_{A}^{i}-\min C_{A}\right)}{\max C_{A}-\min C_{A}},\;\forall S_{A}^{i}\in S_{A},$$

where $\min C_{A}$ and $\max C_{A}$ are the minimum and maximum values in $C_{A}=\left\{C_{A}^{1},C_{A}^{2},\cdots ,C_{A}^{m}\right\}$, respectively.

For the defender, $\alpha_{D}^{j}$ is calculated by

(6) $$\alpha_{D}^{j}=\frac{\theta_{D}\left(C_{D}^{j}-\min C_{D}\right)}{\max C_{D}-\min C_{D}},\;\forall S_{D}^{j}\in S_{D},$$

where $\min C_{D}$ and $\max C_{D}$ are the minimum and maximum values in $C_{D}=\left\{C_{D}^{1},C_{D}^{2},\cdots ,C_{D}^{n}\right\}$, respectively.

Additionally, we propose incorporating an entropy-based measure to quantify the uncertainty and variability of the strategy selection probabilities. The entropy *H* of the attack and defense strategies can be defined as follows:

(7) $$H_{A}=-\sum_{i}P_{A}^{i}\log P_{A}^{i},$$

and

(8) $$H_{D}=-\sum_{j}P_{D}^{j}\log P_{D}^{j}.$$

These entropy measures provide additional insights into the diversity and unpredictability of these strategies. They serve as a metric for assessing the diversity and unpredictability of these strategies.

### 2.3. Payoffs

In Section 2.1, we assume that node $V_{i}$ is successfully removed only if it is attacked by the attacker and is not protected by the defender. We define the sets of removed nodes and edges as $\hat{V}\subseteq V$ and $\hat{E}\subseteq E$, respectively. Then, the resulting network after its removal can be denoted as $\hat{G}=\left(V-\hat{V},E-\hat{E}\right)$.

Here, it is evident that $\hat{V}=V^{A}-V^{A}\cap V^{D}$. The set of removed nodes $\hat{V}$ is equal to the set of nodes attacked by the attacker $V^{A}$ minus the set of nodes attacked by the attacker and protected by the defender $V^{A}\cap V^{D}$. This can be shown by the following calculation:

(9) $$\begin{array}{cc} \hat{V} & =\{V_{i}\in V|V_{i}\in V^{A}\;\mathrm{and}\;V_{i}\notin V^{D}\}\\ \; & =\{V_{i}\in V^{A}|V_{i}\notin V^{D}\}\\ \; & =V^{A}-V^{D}\\ \; & =V^{A}-\left(V^{A}\cap V^{D}\right). \end{array}$$

We denote the measure of network performance as Γ, which can be evaluated by the size of the largest connected component [41], efficiency [42], and other metrics. Additionally, we define the attacker’s payoff as

(10) $$U_{A}^{ij}\left(S_{A}^{i},S_{D}^{j}\right)=\frac{\Gamma \left(G\right)-\Gamma \left(\hat{G}\right)}{\Gamma (G)}\in \left[0,1\right],$$

while the defender’s payoff is defined as

(11) $$U_{D}^{ij}\left(S_{A}^{i},S_{D}^{j}\right)=\frac{\Gamma \left(\hat{G}\right)-\Gamma \left(G\right)}{\Gamma (G)}\in \left[-1,0\right],$$

where Γ is defined as the measure of network performance. In this paper, Γ(G) and $\Gamma (\hat{G})$ are the sizes of the largest connected component of network G(V,E) and network $\hat{G}=\left(V-\hat{V},E-\hat{E}\right)$, respectively. The sum of the attacker’s payoff and the defender’s payoff is zero, indicating a zero-sum game.

## 3. Solution

In a constrained game, the attacker’s objective is to maximize their payoff under strategy constraints, while the defender aims to minimize their loss. Therefore, we establish a linear programming model with two objectives to solve this problem. Let us suppose that z,ω are the expected payoffs for the attacker and the defender, respectively; then, the model is defined as follows:

(12) $$\begin{array}{cc} \max & z\\ \;\;s.t. & \sum_{S_{A}^{i}\in S_{A}}U_{A}\left(S_{A}^{i},S_{D}^{j}\right)\cdot P_{A}^{i}\geq z,\forall S_{D}^{j}\in S_{D}\\ \; & P_{A}^{i}\leq \alpha_{A}^{i},\forall S_{A}^{i}\in S_{A}\\ \; & \sum_{S_{A}^{i}\in S_{A}}P_{A}^{i}=1\\ \; & P_{A}^{i}\geq 0,\forall S_{A}^{i}\in S_{A}, \end{array}$$

(13) $$\begin{array}{cc} \max & \omega \\ \;\;s.t. & \sum_{S_{D}^{j}\in S_{D}}U_{D}\left(S_{A}^{i},S_{D}^{j}\right)\cdot P_{D}^{j}\geq \omega ,\forall S_{A}^{i}\in S_{A}\\ \; & P_{D}^{j}\leq \alpha_{D}^{j},\forall S_{D}^{j}\in S_{D}\\ \; & \sum_{S_{D}^{j}\in S^{D}}P_{D}^{j}=1\\ \; & P_{D}^{j}\geq 0,\forall S_{D}^{j}\in S_{D}, \end{array}$$

where $U_{A}\left(S_{A}^{i},S_{D}^{j}\right)$ is the payoff for the attacker under strategy profile $\left(S_{A}^{i},S_{D}^{j}\right)$ and $U_{D}\left(S_{A}^{i},S_{D}^{j}\right)$ is the payoff for the defender. Equation (12) is the optimization model for the attacker, and Equation (13) is the optimization model for the defender. By solving this model, the Nash equilibrium $\left(P_{A}^{*},P_{D}^{*}\right)$ is obtained. Then, the equilibrium payoff values for the attacker and the defender are defined as

(14) $$z\left(P_{A}^{*},P_{D}^{*}\right)=P_{A}^{T}U_{A}P_{D}=\left(P_{A}^{1},P_{A}^{2},\cdots ,P_{A}^{|S_{A}|}\right)\left(\begin{array}{cccc} u_{A}^{11} & u_{A}^{12} & \cdots & u_{A}^{1|S_{D}|}\\ u_{A}^{21} & u_{A}^{22} & \cdots & u_{A}^{2|S_{D}|}\\ \vdots & \vdots & \; & \vdots \\ u_{A}^{|S_{A}|1} & u_{A}^{|S_{A}|2} & \cdots & u_{A}^{|S_{A}\left|\right|S_{D}|} \end{array}\right)\left(\begin{array}{c} P_{D}^{1}\\ P_{D}^{2}\\ \vdots \\ P_{D}^{|S_{D}|} \end{array}\right),$$

and

(15) $$\omega \left(P_{A}^{*},P_{D}^{*}\right)=P_{A}^{T}U_{D}P_{D}=\left(P_{A}^{1},P_{A}^{2},\cdots ,P_{A}^{|S_{A}|}\right)\left(\begin{array}{cccc} u_{D}^{11} & u_{D}^{12} & \cdots & u_{D}^{1|S_{D}|}\\ u_{D}^{21} & u_{D}^{22} & \cdots & u_{D}^{2|S_{D}|}\\ \vdots & \vdots & \; & \vdots \\ u_{D}^{|S_{A}|1} & u_{D}^{|S_{A}|2} & \cdots & u_{D}^{|S_{A}\left|\right|S_{D}|} \end{array}\right)\left(\begin{array}{c} P_{D}^{1}\\ P_{D}^{2}\\ \vdots \\ P_{D}^{|S_{D}|} \end{array}\right).$$

Due to Equations (10) and (11), we know that this is a zero-sum game, so the payoff for the attacker is equal to the loss of the defender, which is denoted as z=−ω.

## 4. Experiment

In this section, we conducted experiments to demonstrate the effectiveness of our model, using a high-speed rail (HSR) network as an example. In the context of a high-speed rail network, which spans vast geographical areas with numerous stations and control centers, the average distance between nodes plays a pivotal role in the security strategy used. Consider the security strategy for a major HSR network like China’s extensive HSR system, which connects numerous cities across the country. This strategy must ensure the safety and integrity of both passengers and infrastructure. The average distance between stations and control centers is crucial to determine the efficiency and effectiveness of security measures, from real-time monitoring to emergency response coordination.

For comparison purposes, we divided the experiments into two groups: one under unconstrained conditions and the other under constrained conditions. For each group of experiments, we set the number of attackable or defendable nodes to 2, 3, and 4, respectively. We then applied Equations (12) and (13) to generate Nash equilibrium solutions before proceeding with the analysis.

Our analysis was conducted on a system equipped with a 12th Gen Intel Core i7-12700H processor, 32.0 GB of RAM, and a 64-bit operating system running on an x64-based processor. The equipment is from Lenovo, a manufacturer located in Beijing, China. The data originated from the targeted network.

### 4.1. Experiment without Constrained Strategies

#### 4.1.1. Experimental Setting

**Figure 2:**
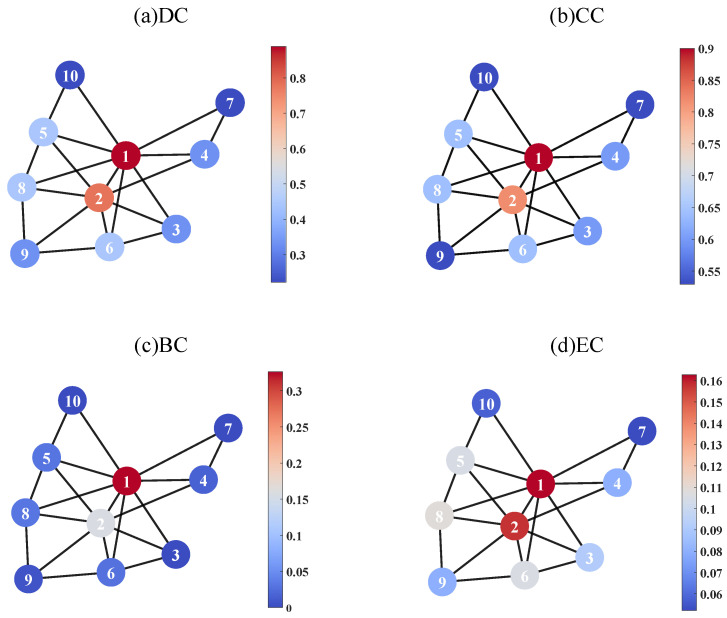
This figure shows the importance of the nodes in terms of various metrics, including degree centrality (DC), closeness centrality (CC), betweenness centrality (BC), and eigenvector centrality (EC). The nodes’ influence is represented by the color of the nodes.

In our experiments conducted within a target network, Figure 2 offers a comprehensive visualization of the nodes’ significance under various centrality metrics: degree centrality (DC), closeness centrality (CC), betweenness centrality (BC), and eigenvector centrality (EC). This visualization employs a color gradient, with the nodes appearing more red having a higher value for the corresponding centrality metric.

Degree centrality (DC ) [43]: This is a measure that quantifies the direct influence of a node on a network. It is based on the principle that nodes with higher degrees have a greater potential to directly affect their neighbors, thereby increasing their significance within the network. The degree of *i* is $k_{i}=\sum_{j=1}^{n}a_{ij}$, which is equal to the number of edges connected to it. This is calculated by

(16) $$\mathrm{DC}\left(i\right)=\frac{k_{i}}{N-1},$$

where *N* denotes the total number of nodes in *G* and N−1 is the maximum possible degree. For normalization, the equation is divided by N-1 based on the degree.

Closeness centrality (CC) [44]: This metric is based on the average time it takes for information to travel from one node to another. It quantifies how quickly a node can reach all other nodes in the network. The closeness centrality of a node is calculated as the sum of the reciprocals of the shortest distances from that node to all other nodes divided by the number of nodes in the network. This value represents the average transmission time needed for information to travel from one node to all other nodes in the network. Nodes with higher closeness centrality values are considered more important because they have greater access to information and can influence the network more quickly. CC is calculated by

(17) $$\mathrm{CC}\left(i\right)=\frac{1}{N-1}\sum_{j\neq i}\frac{1}{d_{ij}},$$

where $d_{ij}$ represents the average shortest distance from node $V_{i}$ to node $V_{j}$. If there is no connection between $V_{i}$ and $V_{j}$, the distance approaches infinity, in which case $\frac{1}{d_{ij}}=\frac{1}{\infty }=0$.

Betweenness centrality (BC) [45]: This is a measure of the influence of a node on the flow of information in a network. This measure quantifies how many shortest paths pass through a particular node and, in turn, how many other nodes are reachable from those paths. The betweenness centrality of a node is calculated by summing the number of shortest paths that pass through each of its neighbors, weighted by the number of shortest paths that include those neighbors. Nodes with higher betweenness centrality values are considered more influential as they play a crucial role in connecting different parts of the network and distributing information efficiently. BC is calculated by

(18) $$\mathrm{BC}\left(i\right)=\frac{2}{N(N-1)}\sum_{s\neq t\neq i}\frac{g_{st}\left(i\right)}{g_{st}},$$

where $g_{st}\left(i\right)$ represents the number of shortest paths from node *s* to node *t* through node *i*. $g_{st}$ represents the total number of shortest paths from node *s* to node *t*.

Eigenvector centrality (EC) [46]: This metric is a measure of the importance of nodes in a network based on the quality of their connections to other nodes. EC quantifies how influential a node is by accounting for not only the number of its neighbors but also the importance of those neighbors. This is calculated by

(19) $$\mathrm{EC}\left(i\right)=\frac{1}{\lambda }\sum_{j=1}^{n}a_{ij}f_{j},$$

where $a_{ij}$ is the adjacency matrix of the network, $f_{j}$ is the value of the *j*th entry of the normalized largest eigenvector, and λ is a constant.

This network consists of 10 nodes and 20 edges. Among the nodes, $V_{1}$ and $V_{2}$ have high values and $V_{7}$ and $V_{10}$ have low values. In this model, a objective function is established based on the size of the largest connected component. We conducted this experiment with different numbers of nodes to be attacked or defended.

#### 4.1.2. The Nash Equilibrium

The mixed-strategy Nash equilibrium results are presented in Table 1, Table 2 and Table 3. Notably, the pure strategies with nonzero probabilities in their equilibrium are listed, as are their respective probabilities. From the equilibrium results when $|V^{A}\left|\;=\;\right|V^{D}|\;=\;2$, we observe that the attacker has five pure strategies with nonzero probabilities. On the one hand, the highest probabilities are assigned to the attack strategies $\left\{V_{3},V_{8}\right\}$, $\left\{V_{4},V_{10}\right\}$, $\left\{V_{5},V_{7}\right\}$, and $\left\{V_{6},V_{9}\right\}$, all of which have a probability of 0.23077. On the other hand, strategy $\left\{V_{1},V_{2}\right\}$ has the lowest probability. Notably, $V_{1}$ and $V_{2}$ have high values for the centrality properties examined in this network. For the defender, strategies $\left\{V_{1},V_{6}\right\}$, $\left\{V_{1},V_{7}\right\}$, $\left\{V_{1},V_{9}\right\}$, and $\left\{V_{2},V_{8}\right\}$ have the highest probabilities, all equal to 0.15385, with $V_{1}$ having the highest value for the centrality properties. When $|V^{A}\left|\;=\;\right|V^{D}|\;=\;3$, the nonzero probabilities of each strategy chosen by both the attacker and defender are given in Table 2. For example, strategy $\left\{V_{2},V_{6},V_{8}\right\}$ has the highest probability among the attacker’s strategies, with a value of 0.2093, and the probability of the defender selecting strategy $\left\{V_{1},V_{6},V_{9}\right\}$ is 0.25. Similarly, in the third scenario, where $|V^{A}\left|\;=\;\right|V^{D}|\;=\;4$, there are a total of eight attack strategies and seven defense strategies with nonzero probabilities. Table 3 provides the probabilities for each strategy. The attacker is more likely to choose strategy $\left\{V_{3},V_{4},V_{6},V_{10}\right\}$, while the defender tends to choose strategies $\left\{V_{1},V_{6},V_{7},V_{9}\right\}$ and $\left\{V_{2},V_{4},V_{5},V_{10}\right\}$.

It is evident that some strategies are much more likely to be chosen than others. For example, in the three scenarios, certain attack and defense strategies have probabilities close to 0.2 or 0.3, respectively. Additionally, by comparing the three scenarios using different values of $|V^{A}|$ and $|V^{D}|$, we can see that the number of nodes to be attacked or defended affects the players’ decision-making results. With an increased number of nodes, players have more flexibility in choosing their strategies, leading to a more complex game.

**Figure 3:**
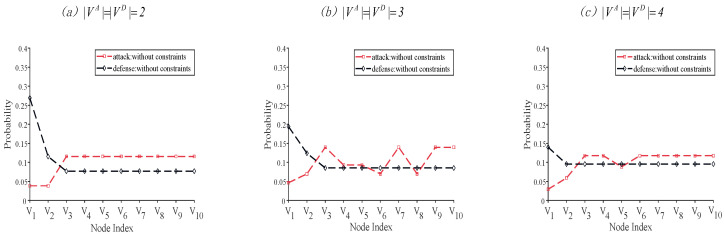
The attack and defense probability distributions for nodes when different numbers of nodes are attacked or defended.

To explore the nodes that the attacker and the defender are most likely to select in the Nash equilibrium, we map the probabilities over pure strategies to those over each node via the following equations:

(20) $$\rho_{A}=\frac{1}{|V^{A}|}\sum_{i=1}^{|S_{A}|}P_{A}^{i}\cdot S_{A}^{i},$$

and

(21) $$\rho_{D}=\frac{1}{|V^{D}|}\sum_{j=1}^{|S_{D}|}P_{D}^{j}\cdot S_{D}^{j},$$

where $\rho_{A}=\left[{\tilde{p}}_{1},{\tilde{p}}_{2},\cdots ,{\tilde{p}}_{i},\cdots ,{\tilde{p}}_{N}\right]$ and $\rho_{D}=\left[{\tilde{q}}_{1},{\tilde{q}}_{2},\cdots ,{\tilde{q}}_{j},\cdots ,{\tilde{q}}_{N}\right]$ are the probability distributions over each node for two players. With this approach, the selection probability distributions for each node are obtained and mapped from the probabilities in Table 1, Table 2 and Table 3, as shown in Figure 3.

The nodes with the lowest probabilities of being attacked are $V_{1}$ and $V_{2}$, whose degree centrality, closeness centrality, betweenness centrality, and eigenvector centrality are the highest. However, the defender allocates the greatest probability to protecting nodes $V_{1}$ and $V_{2}$. This finding suggests that nodes with greater scores are generally more likely to be protected. As the number of nodes to be attacked or defended increases, the probability distribution of the nodes becomes more uniform.

### 4.2. Experiments with Constrained Strategies

#### The Nash Equilibrium

As shown in Section 2.2, $\theta_{A}$ and $\theta_{D}$ in Equations (5) and (6) are determined by various factors. In this experiment, we set $\theta_{A}=0.25$ and $\theta_{D}=0.05$ based on the network structure shown in Figure 2 and the unconstrained Nash equilibrium results in Table 1, Table 2 and Table 3. However, when $|V^{A}\left|\;=\;\right|V^{D}|\;=\;2$, there is no solution for which $\theta_{D}=0.05$. Therefore, when $|V^{A}\left|\;=\;\right|V^{D}|\;=\;2$, we set the critical value $\theta_{D}=0.06$.

Table 4, Table 5 and Table 6 present the mixed-strategy Nash equilibrium results with strategy constraints for scenarios where the numbers of attack and defense nodes are equal ($|V^{A}\left|\;=\;\right|V^{D}|\;=\;2,3,4$). These tables show the first ten highest probabilities of each attack and defense strategy being chosen by both players.

When $|V^{A}\left|\;=\;\right|V^{D}|\;=\;2$, compared to the result without any constraints, it is obvious that both the attacker and the defender are more likely to choose strategies $\left\{V_{1},V_{2}\right\}$ and $\left\{V_{1},V_{5}\right\}$. For the attacker, the probability of selecting strategy $\left\{V_{1},V_{2}\right\}$ is 0.25, and the probability of choosing $\left\{V_{1},V_{5}\right\}$ is 0.20602. For the defender, the probabilities of selecting $\left\{V_{1},V_{2}\right\}$ or $\left\{V_{1},V_{5}\right\}$ are 0.06 or 0.049444, respectively.

When $|V^{A}\left|\;=\;\right|V^{D}|\;=\;3,4$, the probability distribution becomes more uniform. Certain strategies share equal probabilities. For instance, when $|V^{A}\left|\;=\;\right|V^{D}|\;=\;3$, the probabilities of the attacker choosing attack strategies $\left\{V_{6},V_{8},V_{9}\right\}$, $\left\{V_{1},V_{3},V_{4}\right\}$, $\left\{V_{1},V_{4},V_{5}\right\}$, and so on, are all 0.048188. Similarly, the probabilities of the defender selecting defense strategies $\left\{V_{1},V_{2},V_{4}\right\}$, $\left\{V_{1},V_{2},V_{3}\right\}$, $\left\{V_{1},V_{2},V_{5}\right\}$, and so on, are all 0.05. When $|V^{A}\left|\;=\;\right|V^{D}|\;=\;4$, the probability of the attacker choosing attack strategy $\left\{V_{1},V_{2},V_{5},V_{9}\right\}$ is 0.035504, and the probability of them choosing strategies $\left\{V_{3},V_{6},V_{8},V_{9}\right\}$, $\left\{V_{1},V_{3},V_{4},V_{10}\right\}$, and so on, is 0.024047. Similarly, the probability of the defender selecting defense strategies $\left\{V_{1},V_{2},V_{3},V_{6}\right\}$, $\left\{V_{1},V_{2},V_{3},V_{4}\right\}$, $\left\{V_{1},V_{2},V_{4},V_{7}\right\}$, and so on, is 0.020088.

### 4.3. The Probability Distribution of Each Node

**Figure 4:**
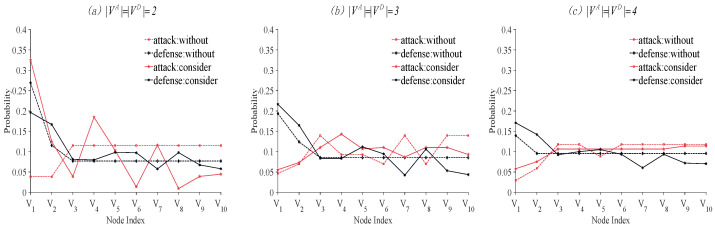
The probabilities of each of the ten nodes being selected by the attacker or the defender, with strategy constraints, are compared with those without constraints when different numbers of nodes are being attacked or defended ($|V^{A}\left|\;=\;\right|V^{D}|\;=\;2,3,4$).

Subsequently, we obtain the distribution of probability across nodes based on Equations (20) and (21). To analyze the various constraints effectively, we have illustrated them in Figure 4.

According to Figure 4, by applying the proposed model, the selection probability of the 10 nodes in the target network changes substantially. When the number of attackable or defendable nodes is two, the change in the selection probability for different nodes is significant. However, as the number of attackable or defendable nodes increases, this change becomes less apparent. Specifically, when the number of nodes to be attacked or defended is two, the selection probabilities $V_{1}$ and $V_{2}$ significantly increase for the attacker. For the defender, the selection probability of $V_{1}$ decreases, while the selection probability of the other nodes does not change significantly. When the number of attackable or defendable nodes is three, there is a small fluctuation in the selection probability of $V_{3},V_{4},V_{5},\cdots ,V_{10}$ for both the attacker and the defender. When the number of nodes is four, only small changes occur.

According to our experiments, we have found key insights that set apart unconstrained and constrained scenarios. Constraints significantly impact the choices of both attackers and defenders. This shows that constraints are not just theoretical; they affect real-world security strategies. Without constraints, decision makers focus on single node metrics when choosing strategies. But with constraints, they must think broadly, considering node interconnections and dependencies. This broadens the strategic landscape, mirroring the complexity of actual security situations.

## 5. Conclusions

Currently, infrastructure attack and defense scenarios have attracted considerable attention. The integration of complex network theory and game theory has provided valuable insights for choosing attack and defense strategies. Modeling an attacker–defender game helps in the analysis of strategic choices. To fit this to realistic situations, we propose a strategy constraint rule and a static game model under this rule.

This approach provides foundational understanding but is recognized to be a simplification of complex realities. In practice, strategic choices are subject to a multitude of constraints, including, but not limited to, resource limitations, temporal dynamics, and regulatory frameworks. The interplay of these factors requires a more integrated model. Future work will involve the development of a more adaptive algorithm. Therefore, we propose several perspectives for future research:

(1) Dynamic constraints: Real-world infrastructure systems are dynamic and constantly changing. Decision makers may face varying constraints over time due to factors such as resource availability, changes in the threat landscape, or evolving regulations. Future research may include exploring the implications of dynamic constraints on the game model and considering how decision makers adapt their strategies based on evolving constraints.

(2) Multiobjective optimization: In addition to constraints, decision makers often need to consider multiple objectives when selecting strategies for infrastructure protection. These objectives may include minimizing damage, maximizing system resilience, or optimizing resource allocation. Future research may include integrating multiobjective optimization techniques into game models to assist decision makers in selecting strategies that balance multiple competing objectives under constrained conditions.

## Figures and Tables

**Table 1:** The mixed-strategy Nash equilibrium results without constraints ($|V^{A}\left|\;=\;\right|V^{D}|\;=\;2$).

Attack strategy	$\left\{V_{3},V_{8}\right\}$	$\left\{V_{4},V_{10}\right\}$	$\left\{V_{5},V_{7}\right\}$	$\left\{V_{6},V_{9}\right\}$	$\left\{V_{1},V_{2}\right\}$
Probability	0.23077	0.23077	0.23077	0.23077	0.076923
Defense strategy	$\left\{V_{1},V_{6}\right\}$	$\left\{V_{1},V_{7}\right\}$	$\left\{V_{1},V_{9}\right\}$	$\left\{V_{2},V_{8}\right\}$	$\left\{V_{4},V_{10}\right\}$
Probability	0.15385	0.15385	0.15385	0.15385	0.15385
Defense strategy	$\left\{V_{1},V_{5}\right\}$	$\left\{V_{2},V_{3}\right\}$	$\left\{V_{3},V_{5}\right\}$		
Probability	0.076923	0.076923	0.076923		

**Table 2:** The mixed-strategy Nash equilibrium results without constraints ($|V^{A}\left|\;=\;\right|V^{D}|\;=\;3$).

Attack strategy	$\left\{V_{2},V_{6},V_{8}\right\}$	$\left\{V_{3},V_{7},V_{10}\right\}$	$\left\{V_{7},V_{9},V_{10}\right\}$	$\left\{V_{1},V_{4},V_{5}\right\}$	$\left\{V_{3},V_{4},V_{9}\right\}$
Probability	0.2093	0.18605	0.13953	0.13953	0.093023
Attack strategy	$\left\{V_{3},V_{7},V_{9}\right\}$	$\left\{V_{5},V_{9},V_{10}\right\}$	$\left\{V_{3},V_{4},V_{5}\right\}$		
Probability	0.093023	0.093023	0.046512		
Defense strategy	$\left\{V_{1},V_{6},V_{9}\right\}$	$\left\{V_{1},V_{8},V_{10}\right\}$	$\left\{V_{2},V_{4},V_{7}\right\}$	$\left\{V_{2},V_{3},V_{5}\right\}$	$\left\{V_{3},V_{4},V_{5}\right\}$
Probability	0.25	0.23837	0.14535	0.14535	0.11047
Defense strategy	$\left\{V_{1},V_{2},V_{7}\right\}$	$\left\{V_{2},V_{7},V_{10}\right\}$	$\left\{V_{1},V_{7},V_{8}\right\}$	$\left\{V_{1},V_{6},V_{7}\right\}$	$\left\{V_{1},V_{7},V_{9}\right\}$
Probability	0.063953	0.017442	0.017442	0.005814	0.005814

**Table 3:** The mixed-strategy Nash equilibrium results without constraints ($|V^{A}\left|\;=\;\right|V^{D}|\;=\;4$).

Attack strategy	$\left\{V_{3},V_{4},V_{6},V_{10}\right\}$	$\left\{V_{3},V_{4},V_{9},V_{10}\right\}$	$\left\{V_{6},V_{7},V_{8},V_{9}\right\}$	$\left\{V_{5},V_{7},V_{9},V_{10}\right\}$
Probability	0.19608	0.13725	0.13725	0.13725
Attack strategy	$\left\{V_{1},V_{2},V_{5},V_{8}\right\}$	$\left\{V_{3},V_{6},V_{7},V_{8}\right\}$	$\left\{V_{2},V_{4},V_{5},V_{8}\right\}$	$\left\{V_{3},V_{4},V_{7},V_{8}\right\}$
Probability	0.11765	0.098039	0.078431	0.039216
Attack strategy	$\left\{V_{2},V_{6},V_{7},V_{9}\right\}$	$\left\{V_{4},V_{5},V_{7},V_{9}\right\}$		
Probability	0.039216	0.019608		
Defense strategy	$\left\{V_{1},V_{6},V_{7},V_{9}\right\}$	$\left\{V_{2},V_{4},V_{5},V_{10}\right\}$	$\left\{V_{1},V_{3},V_{8},V_{10}\right\}$	$\left\{V_{2},V_{3},V_{5},V_{8}\right\}$
Probability	0.32353	0.26471	0.11765	0.058824
Defense strategy	$\left\{V_{1},V_{3},V_{7},V_{8}\right\}$	$\left\{V_{3},V_{6},V_{8},V_{9}\right\}$	$\left\{V_{1},V_{3},V_{4},V_{8}\right\}$	$\left\{V_{2},V_{3},V_{4},V_{5}\right\}$
Probability	0.058824	0.058824	0.058824	0.029412
Defense strategy	$\left\{V_{2},V_{4},V_{5},V_{8}\right\}$			
Probability	0.029412			

**Table 4:** The mixed-strategy Nash equilibrium results with strategy constraints ($|V^{A}\left|\;=\;\right|V^{D}|\;=\;2$).

Attack strategy	$\left\{V_{1},V_{2}\right\}$	$\left\{V_{1},V_{5}\right\}$	$\left\{V_{1},V_{4}\right\}$	$\left\{V_{4},V_{7}\right\}$	$\left\{V_{6},V_{7}\right\}$
Probability	0.25	0.20602	0.19518	0.12259	0.027353
Attack strategy	$\left\{V_{4},V_{10}\right\}$	$\left\{V_{3},V_{9}\right\}$	$\left\{V_{3},V_{7}\right\}$	$\left\{V_{3},V_{10}\right\}$	$\left\{V_{4},V_{9}\right\}$
Probability	0.026268	0.025993	0.025858	0.025724	0.025525
Defense strategy	$\left\{V_{1},V_{2}\right\}$	$\left\{V_{1},V_{5}\right\}$	$\left\{V_{1},V_{6}\right\}$	$\left\{V_{1},V_{8}\right\}$	$\left\{V_{1},V_{4}\right\}$
Probability	0.06	0.049444	0.048545	0.048326	0.046842
Defense strategy	$\left\{V_{1},V_{3}\right\}$	$\left\{V_{2},V_{6}\right\}$	$\left\{V_{2},V_{8}\right\}$	$\left\{V_{2},V_{5}\right\}$	$\left\{V_{1},V_{7}\right\}$
Probability	0.045837	0.045641	0.045254	0.044132	0.04255

**Table 5:** The mixed-strategy Nash equilibrium results with strategy constraints ($|V^{A}\left|\;=\;\right|V^{D}|\;=\;3$).

Attack strategy	$\left\{V_{6},V_{8},V_{9}\right\}$	$\left\{V_{1},V_{3},V_{4}\right\}$	$\left\{V_{1},V_{4},V_{5}\right\}$	$\left\{V_{1},V_{4},V_{10}\right\}$	$\left\{V_{2},V_{4},V_{7}\right\}$
Probability	0.048188	0.048188	0.048188	0.048188	0.048188
Attack strategy	$\left\{V_{2},V_{6},V_{8}\right\}$	$\left\{V_{5},V_{8},V_{9}\right\}$	$\left\{V_{5},V_{8},V_{10}\right\}$	$\left\{V_{3},V_{6},V_{9}\right\}$	$\left\{V_{2},V_{3},V_{9}\right\}$
Probability	0.048188	0.048188	0.048188	0.048188	0.040862
Defense strategy	$\left\{V_{1},V_{2},V_{4}\right\}$	$\left\{V_{1},V_{2},V_{3}\right\}$	$\left\{V_{1},V_{2},V_{5}\right\}$	$\left\{V_{1},V_{2},V_{6}\right\}$	$\left\{V_{1},V_{3},V_{6}\right\}$
Probability	0.05	0.05	0.05	0.05	0.05
Defense strategy	$\left\{V_{2},V_{5},V_{8}\right\}$	$\left\{V_{1},V_{2},V_{8}\right\}$	$\left\{V_{1},V_{4},V_{7}\right\}$	$\left\{V_{1},V_{5},V_{8}\right\}$	$\left\{V_{1},V_{5},V_{10}\right\}$
Probability	0.05	0.05	0.05	0.05	0.05

**Table 6:** The mixed-strategy Nash equilibrium results with strategy constraints ($|V^{A}\left|\;=\;\right|V^{D}|\;=\;4$).

Attack strategy	$\left\{V_{1},V_{2},V_{5},V_{9}\right\}$	$\left\{V_{3},V_{6},V_{8},V_{9}\right\}$	$\left\{V_{1},V_{3},V_{4},V_{10}\right\}$	$\left\{V_{1},V_{3},V_{7},V_{10}\right\}$
Probability	0.035504	0.024047	0.024047	0.024047
Attack strategy	$\left\{V_{5},V_{6},V_{8},V_{9}\right\}$	$\left\{V_{1},V_{4},V_{6},V_{10}\right\}$	$\left\{V_{1},V_{4},V_{8},V_{10}\right\}$	$\left\{V_{2},V_{3},V_{6},V_{7}\right\}$
Probability	0.024047	0.024047	0.024047	0.024047
Attack strategy	$\left\{V_{1},V_{3},V_{4},V_{5}\right\}$	$\left\{V_{2},V_{6},V_{8},V_{9}\right\}$		
Probability	0.024047	0.014469		
Defense strategy	$\left\{V_{1},V_{2},V_{3},V_{6}\right\}$	$\left\{V_{1},V_{2},V_{3},V_{4}\right\}$	$\left\{V_{1},V_{2},V_{4},V_{7}\right\}$	$\left\{V_{1},V_{2},V_{4},V_{8}\right\}$
Probability	0.05	0.020088	0.020088	0.020088
Defense strategy	$\left\{V_{1},V_{2},V_{6},V_{9}\right\}$	$\left\{V_{1},V_{2},V_{8},V_{9}\right\}$	$\left\{V_{1},V_{5},V_{8},V_{10}\right\}$	$\left\{V_{1},V_{2},V_{4},V_{5}\right\}$
Probability	0.020088	0.020088	0.020088	0.020088
Defense strategy	$\left\{V_{1},V_{2},V_{5},V_{10}\right\}$	$\left\{V_{2},V_{5},V_{8},V_{9}\right\}$		
Probability	0.020088	0.020088		

## Data Availability

The data presented in this study are available on request from the corresponding author. The data are not publicly available for privacy reasons.
